# Differential Interactions of the Autonomous Pathway RRM Proteins and Chromatin Regulators in the Silencing of Arabidopsis Targets

**DOI:** 10.1371/journal.pone.0002733

**Published:** 2008-07-16

**Authors:** Isabel Bäurle, Caroline Dean

**Affiliations:** Department of Cell and Developmental Biology, John Innes Centre, Norwich, United Kingdom; Purdue University, United States of America

## Abstract

We have recently shown that two proteins containing RRM-type RNA-binding domains, FCA and FPA, originally identified through their role in flowering time control in Arabidopsis, silence transposons and other repeated sequences in the Arabidopsis genome. In flowering control, FCA and FPA function in the autonomous pathway with conserved chromatin regulators, the histone demethylase FLD and the MSI1-homologue FVE, a conserved WD-repeat protein found in many chromatin complexes. Here, we investigate how the RRM proteins interact genetically with these chromatin regulators at a range of loci in the Arabidopsis genome. We also investigate their interaction with the DNA methylation pathway. In several cases the RRM protein activity at least partially required a chromatin regulator to effect silencing. However, the interactions of the autonomous pathway components differed at each target analysed, most likely determined by certain properties of the target loci and/or other silencing pathways. We speculate that the RNA-binding proteins FCA and FPA function as part of a transcriptome surveillance mechanism linking RNA recognition with chromatin silencing mechanisms.

## Introduction

A significant fraction of eukaryotic genomes comprises repeated sequences including transposons and retroelements. These sequences are effectively silenced through a number of transcriptional and posttranscriptional pathways involving DNA methylation, small RNAs (sRNA) and histone modifications [1], [2], [3]. Plant DNA is methylated at cytosine bases in the CG, CNG (N is any nucleotide) and CHH (H is A, C or T) contexts [4], [5]. CG methylation is efficiently copied onto the daughter strand after DNA replication whereas non-CG methylation requires an active mechanism to re-establish the methylation following replication. For some loci this involves sRNA and the plant-specific RNA polymerase IV (PolIV) [1], [6]. Efficient silencing therefore paradoxically involves transcription of the locus [7]. We have recently identified an additional Arabidopsis pathway involved in silencing of several endogenous transposons and retroelements through the finding that the RRM-domain proteins FCA and FPA play a role in RNA-mediated silencing of a transgenic hairpin [8]. Although this pathway is distinct from the sRNA-directed DNA methylation pathway, both pathways interact closely in a target-specific manner [8]. This is particularly evident from the analysis of the transgene system that originally identified the additional function for FCA and FPA. There, FCA and FPA are required for sRNA amplification [8].

FCA and FPA were originally identified based on their role in flowering time control [9], [10], [11]. Both proteins promote flowering by down-regulating expression of the gene encoding the MADS-domain protein FLOWERING LOCUS C (FLC), which is the major repressor of flowering in Arabidopsis [12], [13]. FCA and FPA both contain multiple RRM-domains but share no other sequence homology. Flowering is closely aligned with seasonal conditions and most pathways impacting on flowering rely on environmental cues such as temperature and photoperiod (reviewed in [14]). *fca* and *fpa* mutants still respond well to environmental cues and were for this reason put into a group named the autonomous pathway (AP). This group also comprises two chromatin regulators, the putative histone H3 K4 histone demethylase FLOWERING LOCUS D (FLD), which is a homologue of human LSD1, and the MSI1 homologue FVE [15], [16], [17]. FVE is one of five Arabidopsis MSI1-like genes, which are homologous to the eukaryotic MSI1 family of WD40 domain-containing proteins found in several protein complexes acting on chromatin [18]. The autonomous pathway also comprises the homeodomain protein LUMINIDEPENDENS (LD) [19], the K homology-domain protein FLOWERING LATE WITH KH MOTIFS (FLK) [20], [21] - also a putative RNA-binding protein - and FY, a homologue of the *S. cerevisiae* 3′-end processing/ polyadenylation factor Pfs2p [22].

The interactions of the AP components FCA, FY and FLD have been analysed [22], [23]. FCA negatively regulates its own expression through alternative transcript 3′ processing, and this and its regulation of *FLC* requires a physical interaction with FY [22], [24]. FCA also requires the activity of the histone demethylase FLD to down-regulate *FLC*, suggesting an RNA metabolism/processing step triggers chromatin changes at *FLC* [23].

Here, we have continued to investigate the role of the AP in chromatin silencing, and have focused on the functional interactions of the RRM-domain proteins FCA and FPA with the chromatin regulators FLD and FVE. We show that FVE, FLD and the third putative RNA-binding protein, FLK, also play a widespread role in chromatin silencing and that they interact functionally in a target specific manner. We also show that the RRM protein FPA largely acts through the histone demethylase FLD in the silencing of *FLC*, reinforcing the conclusion that RRM-type RNA-binding proteins trigger a chromatin change to effect silencing. We find that the interactions of the RRM proteins and the chromatin regulators are different at each target and we exemplify this by comparing *FLC* and *AtMu1* regulation.

## Results and Discussion

### The RRM-domain protein FPA acts through the histone demethylase FLD to suppress *FLC* expression

**Figure 1:**
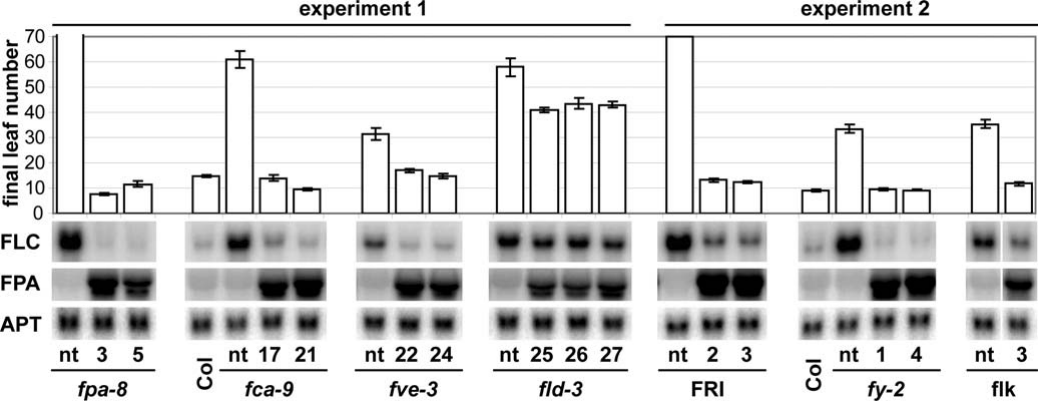
Overexpression of *FPA* in autonomous pathway mutant backgrounds and *FRI*. For each background a non-transformed (nt) control and T2 generation plants from 1–3 independent transformed lines (numbers) were used to assay flowering time and expression levels of *FLC*, *FPA* and *APT* by RNA gel blot analysis. *fpa-8* and *FRI* did not flower during the experiment, which was terminated at ∼70 leaves. Error bars indicate standard error of the mean. Lines were processed in two separate experiments as indicated. Within one experiment, all RNA gel blot panels shown come from the same membrane/ hybridization.

**Figure 2:**
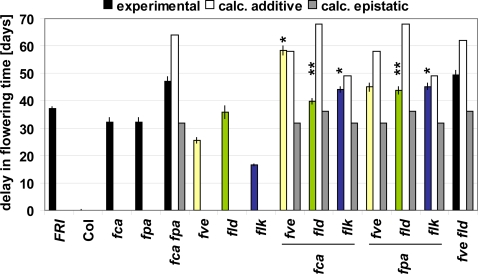
Flowering time of autonomous pathway single and double mutants. Flowering time of the indicated mutant plants grown under long day conditions in a greenhouse was measured in days to flowering (opening of the first flower). Flowering time is indicated as the average delay in flowering relative to the Col wild type +/− standard error of the mean. (yellow (*fve*), green (*fld*), blue (*flk*), and black (all others) bars). Col was flowering at 39 days. White and grey bars indicate predicted flowering time for calculated additive and epistatic scenarios, respectively. *, additive interaction; **, epistatic interaction.

We had previously shown that the RRM protein FCA requires both the 3′ processing/polyadenylation factor FY [22] and the histone demethylase FLD to down-regulate *FLC* [23]. To address whether the second RRM protein, FPA, also requires other AP components for its function, we generated plants expressing *FPA* from a genomic fragment under the control of the constitutive *35S* promoter. The *35S::FPA* construct complemented an *fpa* mutant with respect to *FLC* transcript levels and flowering time and was thus considered fully functional (Figure 1). Overexpression of *FCA* suppresses late flowering and high *FLC* expression levels caused by the presence of the strong *FLC* activator *FRI* [23]. Similarly, we found that overexpression of *FPA* in a *FRI* background repressed *FLC* expression levels and resulted in early flowering, thus confirming that *FPA* overexpression is sufficient to overcome even high *FLC* levels (Figure 1). We then studied whether any of the AP components *FCA*, *FLD*, *FLK*, *FVE*, or *FY* were required for the *FPA*-mediated repression of *FLC*. *FPA* overexpression reduced flowering time and *FLC* levels in *fca*, *flk*, *fve* and *fy* mutant backgrounds to the wild type level, suggesting that these genes are not required for *FPA* function on *FLC*. However, overexpression of *FPA* in an *fld* mutant background reduced both flowering time and *FLC* levels only slightly compared to non-transformed *fld* mutant plants, suggesting that FPA acts in part through FLD. This is further supported by the finding that *fpa fld* double mutants flowered at the same time as the later of the single mutants (Figure 2) and our previous results demonstrating that reactivation of *FLC* in *fpa* and *fld* mutants is at the level of transcription [8], [23]. Together, the finding that the RRM-domain protein FPA represses *FLC* expression through the putative histone demethylase FLD is in line with a model where an RNA-binding component recognizes a particular RNA feature and this triggers chromatin silencing of the locus. Interestingly, while FCA requires both FY and FLD, FPA requires FLD but not FY to repress *FLC*, indicating that the involvement of the histone demethylase FLD is common to both RRM-proteins, while the interaction with the 3′-end processing factor FY is specific to FCA.

### Analysis of flowering time in double mutants

To reveal further interactions between components of the autonomous pathway, we created a number of double mutant combinations in the Columbia (Col) background. We chose Col over the Landsberg *erecta* accession, which had been used for the early genetic work on the autonomous pathway [11], [25], because Landsberg *erecta FLC* carries a transposon insertion in the 3′ end of its first intron and this results in a reduction of expression of the locus through sRNA-mediated silencing [26]. We determined flowering time of the single and double mutants under long days (Figure 2). Previous studies have established that the delay in flowering in AP mutants is caused by the misregulation of *FLC* [13], [15], [27]. All double mutants flowered at least as late as the later single mutant and many considerably later, as expected if independent action of both mutants was assumed. To further analyze the interaction of the studied mutants during flowering time control, we calculated predicted flowering times for the two simplest genetic interactions conceivable and compared them to the experimentally obtained values. If both gene actions were fully independent, we would expect additivity of the delay in flowering in the double mutant (Δ_m1 m2_ = Δ_m1_+Δ_m2_). If one gene action was dependent upon another (epistasis), we would expect the double mutant to flower at the same time as one parent (eg. Δ_m1 m2_ = Δ_m1_, for Δ_m1_>Δ_m2_). This method indicated that *fca fld* have an epistatic interaction and *fca fve* are additive, confirming previous findings [23]. It also indicated *fpa fld* are epistatic and both *fca flk* and *fpa flk* additive, thus complementing the overexpression experiments described above. The flowering time of the remaining double mutants, including *fpa fve*, was intermediate between the two scenarios, suggesting more complex interactions. Thus, in flowering time control, *FCA* and *FPA* both act (at least partly) through the histone demethylase *FLD*, while *FVE* acts independently of *FCA*, but may have a more complex interaction with *FPA*. Finally, the putative RNA-binding protein *FLK* acts independently of both *FCA* and *FPA*.

### Derepression of *AtMu1*, *AtSN1* and *IG/LINE* in double mutants

**Figure 3:**
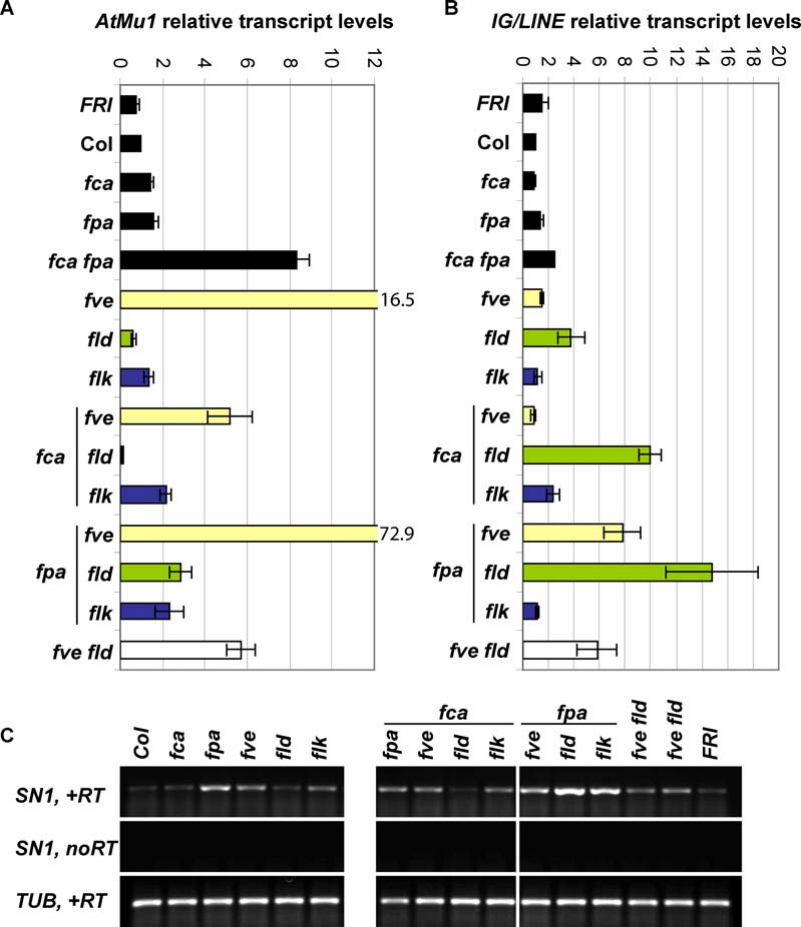
Expression levels of (retro)transposon targets in AP single and double mutants. Expression levels were determined by quantitative RT-PCR for *AtMu1* (A) and *IG/LINE* (B). Data from 3 independent biological replicates were averaged and normalized to Col, +/− standard error of the mean. (C) *AtSN1* expression levels were determined by semi-quantitative RT-PCR (*AtSN1*, 36 cycles; *TUB*, 25 cycles), a representative experiment from 3 independent replicates is shown.

We have recently found that *FCA* and *FPA* also regulate a range of other loci that are subject to sRNA-dependent chromatin silencing [8]. We therefore investigated the interactions of the AP components in their regulation. We first analyzed transcript levels of *AtMu1*, *AtSN1* and *IG/LINE* [6], [28], [29] in *fca*, *fpa*, *fve*, *fld* and *flk* single mutants as well as Col *FRI* plants (Figure 3). While *AtMu1* showed only a slight reactivation of expression in most AP mutants, it was very highly up-regulated in *fve* (16.5 fold over wild type; Figure 3A and [8]). *IG/LINE* expression was only enhanced in *fld* (Figure 3B), whereas *AtSN1* expression was strongly increased in *fpa*, and slightly increased in *fve* and *flk* mutants (Figure 3C). Our previous analysis indicated redundancy between *FCA* and *FPA* in the regulation of these additional targets [8]. However, it was not clear whether this reflected their shared feature of RRM-domains in particular or whether double mutants with other AP components would also show more-than-additive effects. We therefore analyzed the available double mutants in the Col background, focussing on the RRM-domain proteins FCA and FPA and the chromatin regulators FVE and FLD. Indeed, a number of these double mutants showed stronger reactivation of *AtMu1*, *AtSN1* and *IG/LINE* than any of the single mutants. Most noticeably, *fpa fve* showed a 4.5-fold increase in expression of the DNA transposon *AtMu1* over *fve* (73-fold over wild type, Figure 3A). *fca fve* and *fve fld* showed an increase in *AtMu1* expression compared to wild type but less than *fve* alone. The significance of this reduction in these double mutants is at present unclear. *fpa fld* and *fpa flk* both showed slightly higher *AtMu1* expression than any of the respective single mutants. Unexpectedly, *fca fld* mutants (but not *fpa fld* or *fve fld* mutants) consistently showed hyper-repression of *AtMu1* expression (5-10-fold).

*IG/LINE* is an intergenic transcript flanked by a solo LTR which presumably acts as a promoter element [29]. Despite lack of *IG/LINE* reactivation in any of the single mutants with the exception of a slight increase in *fld*, in the majority of double mutants tested *IG/LINE* expression was reactivated (Figure 3B). This indicates a function in *IG/LINE* repression for all the AP mutants tested here, most obviously seen in *fpa fld*, *fca fld* and *fpa fve*.

The retroelement *AtSN1* is reactivated in all double mutants with *fpa*, *fve* or *flk* to an extent which approximately reflects the addition of the reactivation in the respective single mutants (Figure 3C). The one exception was *fpa fld* which displayed a strong synergistic reactivation of *AtSN1*. Thus, the most obvious conclusion is that *FPA*, *FVE*, *FLD* and *FLK* act largely independently on *AtSN1*.

These data therefore reveal the inherent redundancy of AP components. Effects on the targets are in most cases only revealed in double mutant backgrounds and the variation at the different loci presumably reflects their differential interaction with each other and with other silencing pathways.

### Differential interactions of FCA, FPA and FVE on *FLC* and *AtMu1*

**Figure 4:**
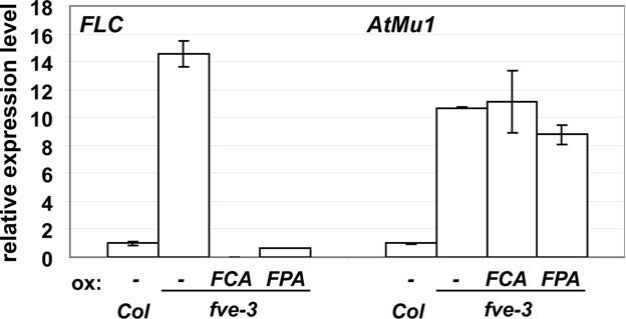
Interaction of *FCA*, *FPA* and *FVE* on *FLC* and *AtMu1*. Overexpression (ox) of *FCA* or *FPA* compensated loss of *FVE* on *FLC*, but not *AtMu1*. *FLC* and *AtMu1* expression in the indicated genotypes were determined by quantitative RT-PCR. Error bars indicate standard error of the mean.

To further dissect the differential interactions between AP components, we analyzed the effect of *FCA*, *FPA* and *FVE* on *AtMu1* regulation in more detail and compared it to the situation at *FLC*. At the *FLC* locus, overexpression of either *FCA* or *FPA* can compensate for the loss of FVE protein and reduce *FLC* expression to or below wild type levels (Figure 4), suggesting that both FCA and FPA act independently of FVE on *FLC*. The strong reactivation of *AtMu1* in *fve* enabled us to ask whether the same was true for *AtMu1*. Overexpression of *FCA* or *FPA* in an *fve* mutant did not restore silencing of *AtMu1* (Figure 4), suggesting that either FCA and FPA act through FVE on *AtMu1*, or that FCA and FPA act in parallel to FVE and overactivation of FCA or FPA is not sufficient to counteract loss of FVE. *fca fve* did not show higher *AtMu1* expression than *fve* (Figure 3A), consistent with the notion that FCA is working through FVE on *AtMu1*. By contrast, *fpa fve* did show higher *AtMu1* expression than *fve*, consistent with independent action of both genes. Furthermore, the *fve* mutant background was more sensitive (than wild type) to loss of *fpa* with respect to *AtMu1* reactivation, highlighting the idea of redundancy between different AP components.

### The autonomous pathway mediates silencing through DNA methylation-dependent and –independent effects

**Figure 5:**
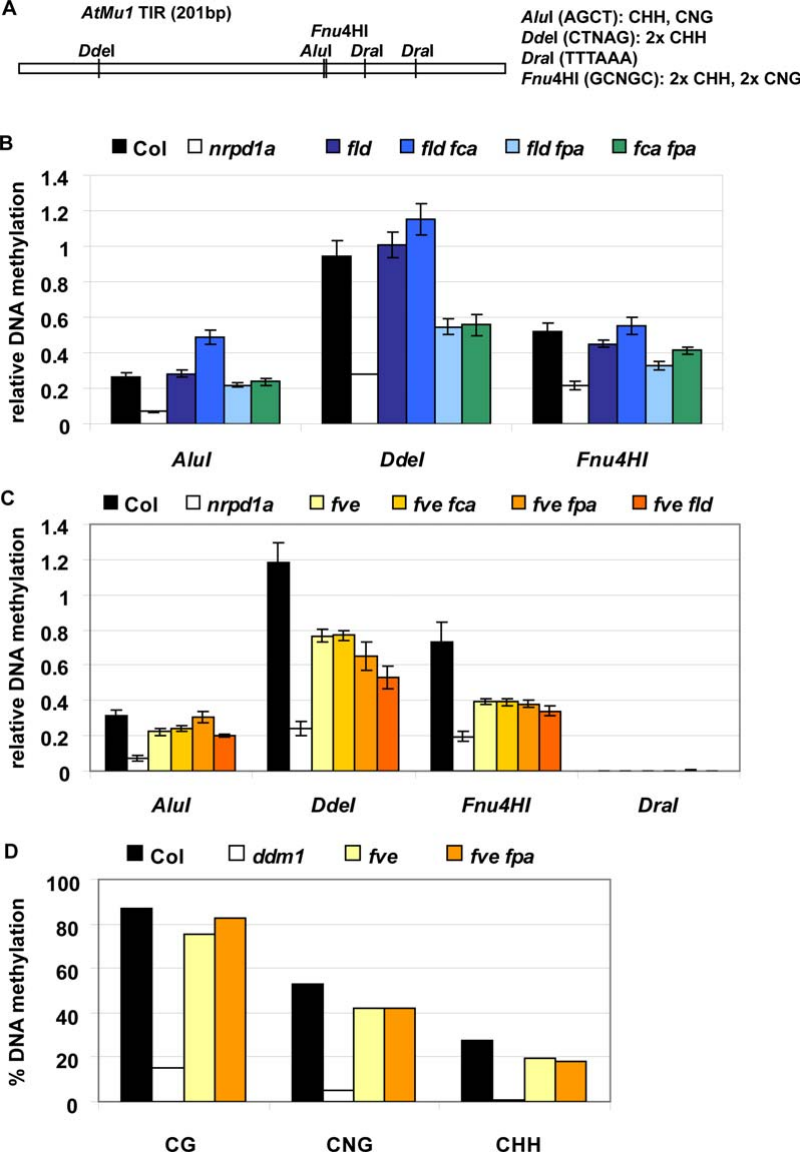
DNA methylation at *AtMu1* in double mutants. (A) Schematic representation of the *AtMu1* TIR showing the recognition sites of the enzymes used in (B) and (C), and the type of methylation analyzed. *Dra*I was used as a control for complete digestion in all experiments ((C) and data not shown). (B) and (C) Relative DNA methylation assayed by quantitative PCR of restriction enzyme digested DNA. Error bars indicate standard error of the mean. (D) Bisulfite sequencing of *AtMu1* TIR; *ddm1*, in which DNA methylation is strongly decreased [28], was included as a control.

Silencing of *AtMu1* is associated with both symmetric (CG) and asymmetric (CNG, CHH) DNA methylation [28], [30]. Derepression of *AtMu1* in *fca fpa* correlated with a loss in asymmetric DNA methylation ([8] and Figure 5B). To address whether a similar loss in DNA methylation at *AtMu1* occurred in other AP mutants with *AtMu1* mis-regulation, we analyzed DNA methylation of the Terminal Inverted Repeats (TIRs). First, we used an assay that combines digestion of DNA using DNA methylation-sensitive restriction enzymes and quantitative PCR (Figure 5A). As controls, we used the methylation-insensitive enzyme *DraI* and compared the mutants to *nrpd1a* (PolIVa) mutants, in which most of the asymmetric DNA methylation is lost [6], [8]. Using three different enzymes that report on CNG and CHH sites (Figure 5A), we found a pronounced loss of DNA methylation in *fve* and in *fve fca*, *fve fpa*, *fve fld*, and *fld fpa* (Figure 5B, C). Despite the stronger reactivation of *AtMu1* expression in *fve fpa* compared to *fve*, DNA methylation levels in both mutants were similarly low (Figure 5C), suggesting that the further increase in expression was independent of DNA methylation. We confirmed the loss of DNA methylation at CNG and CHH sites in *fve* and *fve fpa* using bisulfite sequencing of the *AtMu1* TIR region (Figure 5D, Table S1). CG DNA methylation at *AtMu1* was not or only slightly affected in *fve* and *fpa fve*.

During the double mutant expression analysis, we found that *fca fld* mutants had hyper-repressed *AtMu1*. Interestingly, this hyper-repression correlated with an increase in asymmetric DNA methylation in *fca fld*, but not *fld* single mutants (Figure 5B). Further studies will be necessary to understand the basis of this effect. At present, we can speculate that in the absence of FLD, an FLD-like protein can take its place; in the presence of FCA, this FLD-like protein would contribute to basal activation of AtMu1, whereas loss of FCA would cause this protein to become a strong repressor, possibly by switching its specificity to demethylate certain residues on histone tails. FLD homologues have been described recently [31], as has the context-dependent switch of specificity for the human FLD homologue, LSD1 [32], [33].

**Figure 6:**
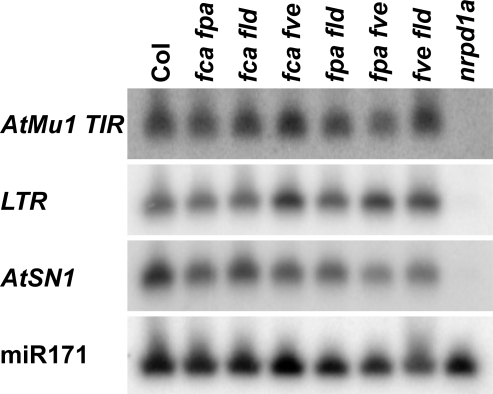
Accumulation of sRNA from a range of targets (*AtMu1*, *AtSN1*, *IG/LINE* (solo LTR)) is not affected in AP double mutants. 9 µg of RNA enriched for the low molecular weight fraction from 14 day old seedlings of the indicated double mutants or *nrpd1a* was loaded per lane. Micro RNA miR171 is shown as a control.

Asymmetric DNA methylation is thought to be directed by sRNA [1], [3], [5]. We did not find a change in the abundance of sRNA at *AtMu1*, *AtSN1* or *IG/LINE (soloLTR)* in any of the double mutants tested (Figure 6), suggesting that none of the AP genes play a role in the amplification of sRNA, but rather that they act either downstream or independent of sRNA. *AtMu1* sRNA and asymmetric DNA methylation are lost in *PolIVa* mutants, yet expression increases only about 6-fold [8]. In contrast, we have shown here that *AtMu1* expression in *fve fpa* increases ∼70-fold, suggesting the involvement of DNA methylation-independent effects besides the observed reduction in DNA methylation.

**Figure 7:**
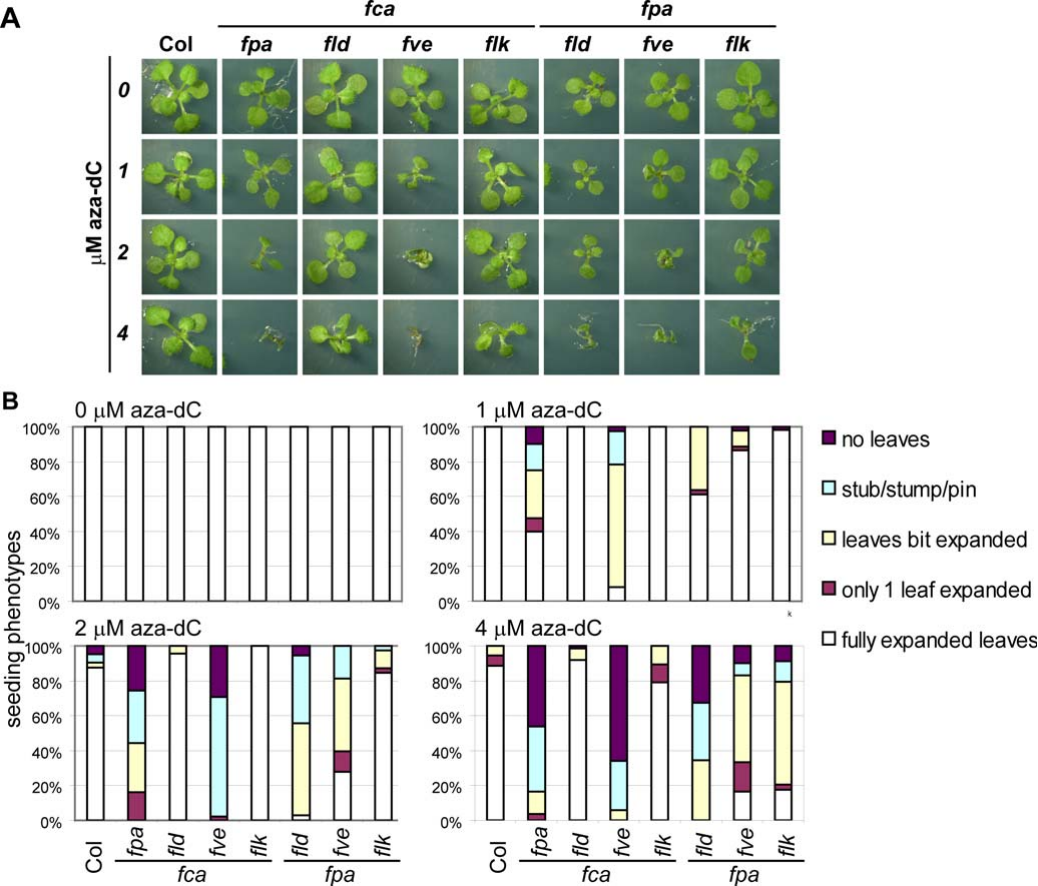
Several AP double mutants (*fca fpa*, *fca fve*, *fpa fld*, *fpa fve*, *fpa flk*) are hypersensitive to the DNA methylation inhibitor aza-dC. Seedlings were grown for 14 days on plates containing the indicated concentration of aza-dC before their phenotypes were scored. (A) A representative seedling of the indicated genotypes and treatments is shown. All pictures are the same magnification and represent 15 mm×15 mm original size. (B) Seedlings were grouped into different classes based on the phenotype of their primary leaves (fully expanded leaves, only 1 leaf expanded, leaves bit expanded stub/stump/pin, no leaves). Severity of the phenotypes increased with increasing aza-dC concentration.

**Figure 8:**
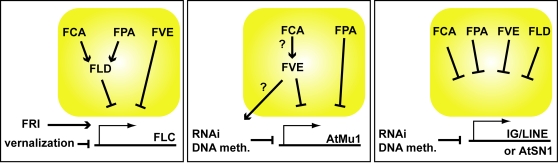
The RRM protein (FCA, FPA) and the chromatin regulator (FLD, FVE) components of the AP interact differently at different targets. At *FLC*, *FCA* acts at least partially through *FLD*, *FPA* acts at least partially through *FLD* and *FVE* acts independently. *FCA* potentially acts through *FVE* to silence *AtMu1*, while *FPA* acts independently. *FCA*, *FPA*, *FLD* and *FVE* all act independently to silence expression of *IG/LINE* and *AtSN1*.

Reactivation of transcription in the presence of DNA methylation has previously been reported for the targets of the *MORPHEUS' MOLECULE1* (*MOM1*) gene [34], [35] and for *AtSN1* in *fca fpa* [8]. Both *MOM1* and *FCA FPA* act in parallel to DNA methylation, and loss of DNA methylation through mutation or application of the DNA methylation inhibitor 5-aza-deoxycytidine (aza-dC) in *mom1* or *fca fpa* leads to dramatic developmental perturbations [8], [34]. To find evidence for the DNA methylation-independent role of other components of the autonomous pathway, we tested whether any of the double mutants tested in this study showed hypersensitivity to aza-dC. Indeed, at aza-dC concentrations that did not affect development in wild type or *fca* or *fpa* single mutants, development in *fca fve* and *fpa fld* mutant seedlings was strongly perturbed similar to what was reported for *fca fpa* (Figure 7, Table S2 and [8]). *fpa fve* and *fpa flk* were also hypersensitive to aza-dC, albeit to a slightly lesser extent (Figure 7). Together, our results demonstrate that AP components mediate silencing through both DNA methylation-dependent and -independent effects (Figure 8).

### Conclusions

The autonomous pathway was initially identified as a flowering-specific pathway that promotes flowering by repressing expression of the floral repressor *FLC*. However, it is now clear that it has more widespread roles on other targets in the Arabidopsis genome [8], [36]. Here, we have investigated how components of the autonomous pathway functionally interact to achieve this silencing.

Using gain-of-function analysis of the RRM-domain protein FPA to complement our loss-of-function double mutant analysis, we find that FPA at least partially acts through the histone demethylase FLD to repress *FLC*. Notably, FPA acts independently of the 3′-processing factor FY. This is in contrast to the other RRM-domain protein FCA, which acts through both FY and FLD. Thus, FCA and FPA have similar but distinct functions in repressing *FLC*. The MSI1 homologue FVE, in contrast, functions independently of FCA and FPA on *FLC*. However, analysis of the DNA transposon *AtMu1* in double mutants and lines overexpressing FCA or FPA in an *fve* mutant background is consistent with the notion that on this target FCA acts through FVE.

In general, we find that the effects of the AP genes and their interactions differ with each target analyzed and show no correlation with *FLC* levels, indicating the observed widespread effects are unlikely to be secondary effects of *FLC* overexpression. We therefore view the autonomous pathway not as a linear pathway, but rather a module of proteins whose role may be to recognize certain RNA features (presumably via the RNA-binding proteins) and trigger a reduction in the transcription of the corresponding loci (presumably via the chromatin regulators). This process is likely to be highly coordinated with transcription and transcript maturation (processing, capping, splicing). It is also possible that different modules of the autonomous pathway interact with different parts of the transcription and maturation machinery. We propose that the autonomous pathway is part of a widely conserved transcriptome surveillance mechanism and in *Arabidopsis* the gene encoding the flowering repressor FLC has, perhaps through selection for flowering time variation, become a very sensitive target.

## Materials and Methods

### Plant Materials

All mutants were in the Col background and have been described; *fca-9*, *fpa-7*, *fpa-8* [8], *fve-3* [16], [17], *fld-3*, *fld-4* [15], *flk-1* [20], Col *FRI Sf2* [37], *nrpd1a-5* [38], *ddm1-2* [39]. Plants were grown in long day conditions in soil at 23°C or on GM minus glucose plates at 20°C.

### Construction of *35S::FPA*

A genomic FPA fragment (coding sequence plus introns) was amplified with flanking BamHI sites (primers 30/FPA_BamHI_F (AAAGGATCCACAATGGCGTTATCTATGAAGCCATTCAGAGC) and 31/FPA_BamHI_R (AAAGGATCCTCAAGGCCCCTGTCCAGCCGGAGTA)) and inserted into 35S::pBIN-Plus [40].

### RNA and DNA methylation analysis

RNA was extracted from 14 day old seedlings and analyzed as described [8]. Bisulfite sequencing was performed as described [8]. For determining DNA methylation through quantitative PCR, we extracted DNA from 14 day old seedlings using the QIAGEN DNeasy Plant Mini Kit and digested 20 ng of DNA overnight with 15 units of the indicated restriction enzyme. After inactivating the restriction enzyme, we immediately performed quantitative PCR using 0.3 ng of DNA per PCR reaction and primers 96/MuTIR_F and 97/MuTIR_R as described in [8]. Primers for FLC quantitative RT-PCR were FLC_cDNA_393F (AGCCAAGAAGACCGAACTCA) and FLC_cDNA_550R (TTTGTCCAGCAGGTGACATC). All other primers have been described [8].

## Supporting Information

Table S1 Additional information for Bisulfite sequencing of AtMu1.
(0.04 MB DOC)

Table S2 Additional information on the Percentages of abnormal seedlings after 14d growth on the indicated concentration of aza-dC
(0.10 MB DOC)
