# Supplementary material for: Differential Interactions of the Autonomous Pathway RRM Proteins and Chromatin Regulators in the Silencing of Arabidopsis Targets
Source: PLoS One. 2008 Jul 16;3(7):e2733. doi: 10.1371/journal.pone.0002733 (PMC2444039; doi:10.1371/journal.pone.0002733)
Supplement: Table S1 — Additional information for Bisulfite sequencing of AtMu1. (0.04 MB DOC) [file pone.0002733.s001.doc]

**Table S1. Bisulfite sequencing of *AtMu1*. N is A, C, G or T; H is A, C or T.**

| **AtMu1** |  | **CG** | **CNG** | **CHH** | **number of clones analyzed** |
| --- | --- | --- | --- | --- | --- |
| number of sites in sequence analyzed | | 6 | 5 | 68 |  |
|  |  |  |  |  |  |
| number of sites analyzed | Col | 132 | 110 | 1496 | 22 |
|  | *ddm1-2* | 120 | 100 | 1360 | 20 |
|  | *fve-3* | 102 | 85 | 1156 | 17 |
|  | *fve-3 fpa-7* | 138 | 115 | 1564 | 23 |
|  |  |  |  |  |  |
|  |  |  |  |  |  |
| proportion of methylated C | Col | 0.871 | 0.527 | 0.277 |  |
|  | *ddm1-2* | 0.150 | 0.050 | 0.010 |  |
|  | *fve-3* | 0.755 | 0.424 | 0.197 |  |
|  | *fve-3 fpa-7* | 0.826 | 0.417 | 0.180 |  |
|  |  |  |  |  |  |
