# Supplementary material for: Differential Interactions of the Autonomous Pathway RRM Proteins and Chromatin Regulators in the Silencing of Arabidopsis Targets
Source: PLoS One. 2008 Jul 16;3(7):e2733. doi: 10.1371/journal.pone.0002733 (PMC2444039; doi:10.1371/journal.pone.0002733)
Supplement: Table S2 — Additional information on the Percentages of abnormal seedlings after 14d growth on the indicated concentration of aza-dC (0.10 MB DOC) [file pone.0002733.s002.doc]

**Table S2**. Percentages of abnormal seedlings after 14d growth on the indicated concentration of aza-dC.

| aza-dC  (μM) | genotype | leaf phenotype (%) | | | | |  |
| --- | --- | --- | --- | --- | --- | --- | --- |
|  |  | fully expanded | only one leaf expanded | bit expanded | stub/stump/pin | no leaves | n |
| 0 | Col | 100.0 | 0.0 | 0.0 | 0.0 | 0.0 | 42 |
|  | *fca fpa* | 100.0 | 0.0 | 0.0 | 0.0 | 0.0 | 36 |
|  | *fca fld* | 100.0 | 0.0 | 0.0 | 0.0 | 0.0 | 29 |
|  | *fca fve* | 100.0 | 0.0 | 0.0 | 0.0 | 0.0 | 28 |
|  | *fca flk* | 100.0 | 0.0 | 0.0 | 0.0 | 0.0 | 40 |
|  | *fpa fld* | 100.0 | 0.0 | 0.0 | 0.0 | 0.0 | 42 |
|  | *fpa fve* | 100.0 | 0.0 | 0.0 | 0.0 | 0.0 | 52 |
|  | *fpa flk* | 100.0 | 0.0 | 0.0 | 0.0 | 0.0 | 48 |
|  |  |  |  |  |  |  |  |
|  |  |  |  |  |  |  |  |
| 1 | Col | 100.0 | 0.0 | 0.0 | 0.0 | 0.0 | 46 |
|  | *fca fpa* | 40.0 | 7.5 | 27.5 | 15.0 | 10.0 | 40 |
|  | *fca fld* | 100.0 | 0.0 | 0.0 | 0.0 | 0.0 | 57 |
|  | *fca fve* | 8.1 | 0.0 | 70.3 | 18.9 | 2.7 | 37 |
|  | *fca flk* | 100.0 | 0.0 | 0.0 | 0.0 | 0.0 | 50 |
|  | *fpa fld* | 61.1 | 2.8 | 36.1 | 0.0 | 0.0 | 36 |
|  | *fpa fve* | 86.4 | 2.3 | 9.1 | 0.0 | 2.3 | 44 |
|  | *fpa flk* | 98.1 | 0.0 | 0.0 | 0.0 | 1.9 | 53 |
|  |  |  |  |  |  |  |  |
|  |  |  |  |  |  |  |  |
| 2 | Col | 87.5 | 0.0 | 3.1 | 4.7 | 4.7 | 64 |
|  | *fca fpa* | 0.0 | 16.3 | 27.9 | 30.2 | 25.6 | 43 |
|  | *fca fld* | 95.5 | 0.0 | 4.5 | 0.0 | 0.0 | 44 |
|  | *fca fve* | 0.0 | 2.1 | 0.0 | 68.8 | 29.2 | 48 |
|  | *fca flk* | 100.0 | 0.0 | 0.0 | 0.0 | 0.0 | 56 |
|  | *fpa fld* | 2.8 | 0.0 | 52.8 | 38.9 | 5.6 | 36 |
|  | *fpa fve* | 27.9 | 11.6 | 41.9 | 18.6 | 0.0 | 43 |
|  | *fpa flk* | 84.6 | 2.6 | 10.3 | 2.6 | 0.0 | 39 |
|  |  |  |  |  |  |  |  |
|  |  |  |  |  |  |  |  |
| 4 | Col | 88.7 | 5.7 | 5.7 | 0.0 | 0.0 | 53 |
|  | *fca fpa* | 0.0 | 3.7 | 13.0 | 37.0 | 46.3 | 54 |
|  | *fca fld* | 91.9 | 0.0 | 6.5 | 0.0 | 1.6 | 62 |
|  | *fca fve* | 0.0 | 0.0 | 6.0 | 28.0 | 66.0 | 50 |
|  | *fca flk* | 78.9 | 10.5 | 10.5 | 0.0 | 0.0 | 38 |
|  | *fpa fld* | 0.0 | 0.0 | 34.6 | 32.7 | 32.7 | 52 |
|  | *fpa fve* | 16.7 | 16.7 | 50.0 | 6.7 | 10.0 | 30 |
|  | *fpa flk* | 17.6 | 2.9 | 58.8 | 11.8 | 8.8 | 34 |
